# A slow-release dexamethasone implant for cancer-associated retinopathy

**DOI:** 10.5935/0004-2749.20230006

**Published:** 2022-01-31

**Authors:** Leandro J. Chaves, Marina L. de Albuquerque, Amanda Schnorr, Francyne Cyrino, Maria Lúcia Habib, André Messias, Zelia M. Correa, Rodrigo Jorge

**Affiliations:** 1 Department of Ophthalmology, Otorhinolaryngology and Head and Neck Surgery Faculdade de Medicina de Ribeirão Preto, Universidade de São Paulo, Ribeirão Preto, SP, Brazil; 2 Ocular Oncology Service, Bascom Palmer Eye Institute and Sylvester Cancer Center, University of Miami, Miami, FL, USA

**Keywords:** Retinopathy, Dexamethasone, Paraneoplastic ocular syndromes, Electroretinography, Retinopatia, Dexametasona, Síndromes paraneoplásicas oculares, Eletroretinografia

## Abstract

This case report aims to show the anatomical and functional results of a patient diagnosed as having cancer-associated retinopathy treated with a controlled-release dexamethasone implant (Ozurdex). Anatomical outcomes were assessed using spectral domain optical coherence tomography; and functional outcomes, by measuring visual acuity, microperimetry, and mutifocal electroretinography. The follow-up period was 1 year.

## Abstract (pt)

Este relato de caso tem como objetivo mostrar os resultados anatômicos e funcionais de um paciente com diagnóstico de retinopatia associada ao câncer tratado com implante de liberação controlada de dexametasona (Ozurdex®). Os resultados anatômicos foram avaliados por SD-OCT e os resultados funcionais por medida de acuidade visual, microperimetria e eletrorretinograma multifocal. O período de acompanhamento foi de um ano.

## INTRODUCTION

Paraneoplastic ocular syndromes include different entities such as cancer-associated retinopathy (CAR), melanoma-associated retinopathy, and bilateral diffuse uveal melanocytic proliferation, among others^(1, 2)^. The neoplasm may trigger a self-aggressive response against healthy autologous tissues distant from the tumor, with no direct relationship with metastases or local invasion^(2)^.

CAR is characterized by bilateral progressive visual loss over periods of weeks that may be asymmetrical or asynchronous. The symptoms are related to the involvement of cones, such as photophobia, photopsia, glare, central scotoma, changes in color vision, and/or rods; night blindness; and loss of a peripheral visual field. The ophthalmologic examination result may be normal or reveal vitritis, arteriolar narrowing, optic disc pallor, and/ or changes of the retinal pigment epithelium, depending on the time course^(1, 2)^.

## CASE REPORT

**Figure 1.**
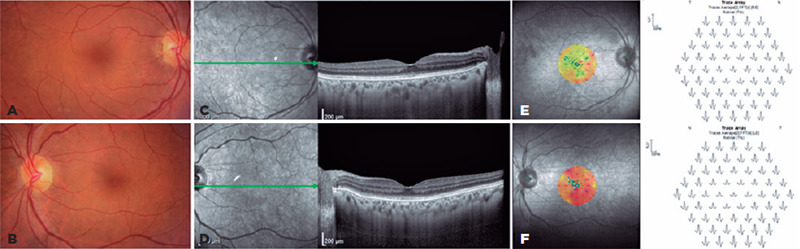
Baseline multimodal assessment. (A, B) An unremarkable color fundus retinography finding. (C, D) Spectral domain optical coherence tomography image showing the granular aspects of the inner and outer segment layers and ellipsoid zone. (E, F) Baseline microperimetry sensitivity map is abnormal in both eyes. (G, H) Multifocal electroretinogram trace array showing an abnormal asymmetrical reduction in central response in both eyes.

A 66-year-old woman had a history of breast cancer treated 13 years earlier with lumpectomy, radiotherapy, and chemotherapy followed by 5 years of tamoxifen therapy. She was in remission, but after an uneventful cataract surgery, she began to complain of photophobia and decreased vision. Her best-corrected visual acuity (BCVA) was 20/100 in the right eye (oculus dexter [OD]) and 20/800 in the left eye (oculus sinister [OS]). Her pre--cataract surgery BCVA was 20/40 in both eyes (oculus uterque [OU]). The ophthalmologic examination result was unremarkable (Figure 1A, B). The retinal nerve fiber layer was normal in OU on optical coherence tomography (OCT), and macular OCT revealed an asymmetrical granular aspect of the inner/outer photoreceptor layer (IS/OS layer) and ellipsoid zone in OU (Figure 1C, D). Microperimetry revealed good fixation stability and abnormal macular integrity in OU. The mean threshold was 21.8 dB for the OD and 12.6 dB for the OS (Figure 1E, F). Multifocal electroretinography (ERG) showed an abnormality in OU, showing an asymmetrical reduction in central response (Figure 1G, H).

The working diagnosis was CAR. As antibody tests were not available and no tumor recurrence was detected in the oncological reevaluation, the patient refused to receive any further systemic treatment. In this context, our group proposed compassionate use of an off-label intravitreal 0.7-mg dexamethasone implant (Ozurdex) that is commercially available as a treatment option for the OS. The patient consented to local treatment for the left eye only.

**Figure 2.**
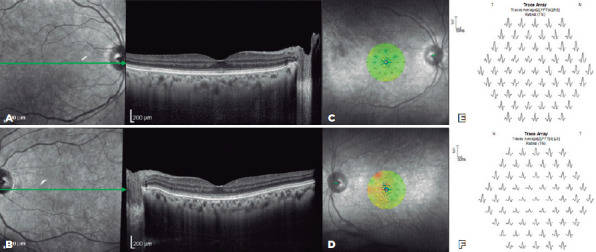
Clinical outcome 30 days after insertion of the controlled-release dexamethasone implant in the left eye. (A) Spectral domain optical coherence tomography (OCT) image showing no changes in the right eye. (B) Spectral domain OCT image showing partial recovery of the inner and outer segment layers and ellipsoid zone of the left eye. (C, D) Microperimetry sensitivity maps showing abnormal macular integrity especially in the left eye, although it is an improvement from that at baseline. (E, F) Abnormal multifocal electroretinogram trace array in both eyes, showing an asymmetrical reduction in central response.

One month post implant placement, the patient reported improvement of visual acuity and resolution of photophobia. Her BCVA was 20/50 in the OD and 20/400 in the OS. The intraocular pressure was normal, and no posterior capsule opacity was observed. Macular spectral domain optical coherence tomography revealed a better definition of the retinal layers, and on microperimetry, a slight increase in mean threshold was identified, up to 26 dB for the OD and 23 dB for the OS. Multifocal ERG failed to detect any changes (Figure 2).

Seven months post implant placement, the patient returned with a recent diagnosis of *de novo* breast cancer (invasive ductal carcinoma) treated with radical mastectomy and chemotherapy. The ophthalmic examination findings remained stable 12 months after the steroid intravitreal implant.

## DISCUSSION

Some antibodies have been related to CAR^(2)^. Among these antibodies is antirecoverin, which crosses the blood-retina barrier and leads to photoreceptor apoptosis. However, the diagnosis cannot be based solely on the antibodies detected because they are detected in the absence of a cancer diagnosis^(3)^. In the present case, no tests for detecting antibodies were available in the local laboratories. However, the patient’s clinical history, symptoms, OCT findings, and ERG findings supported the hypothesis of CAR.

ERG can detect cone and/or rod dysfunction^(1, 2, 4)^. Thus, it should be requested when symptoms do not correlate with the ophthalmologic findings. Full-field ERG results can be normal or show abnormal scotopic responses that indicate global rod dysfunction and/or abnormal photopic responses that indicate global cone dysfunction. Patients may present with central cone dysfunction evident only on multifocal ERG^(4)^. OCT can be normal or reveal irregularity in the IS/OS and ellipsoid layers and macular edema^(5, 6)^.

No strict diagnostic criteria have been established for CAR^(1, 2, 3)^. The diagnosis is based on symptoms, functional and anatomical examination results, a systemic diagnosis of cancer, and presence of antibodies against retinal proteins^(1, 2, 3, 4, 5, 6)^. It is important to exclude retinal degenerations, drug-related retinopathy, hereditary optic neuropathy, and nutritional/toxic neuropathy^(1,2, 3, 4, 7)^. Our patient had a negative family history of retinal degeneration, smoking, and alcohol drinking and did not show any changes suggestive of tamoxifen toxicity.

A suspicion of CAR should promote an early oncological assessment^(1, 2, 3, 4, 5, 6)^. Our patient’s oncological reevaluation did not reveal tumor recurrence, new neoplasia, or metastases immediately after ophthalmic presentation. Thus, we were unsure about the possibility of recurrence of CAR symptoms 13 years after her previous cancer, especially because she had been in remission for over a decade. We speculate that her cataract surgery, with its inherent inflammatory process and the breaking of the blood-retinal barrier, could be involved in the changes detected. Unfortunately, the patient was diagnosed as having *de novo* breast cancer 7 months after the ophthalmic presentation.

Currently, no treatment has been established for CAR. Some authors have proposed that early treatment of the neoplasm and the use of steroids or immunomodulators may change the natural course of the disease^(4, 5, 7)^. Without treatment, most patients progress with a severe visual loss^(4, 5)^, although spontaneous improvement has been reported^(6)^. Different therapeutic modalities have been used anecdotally, including pulse corticosteroid therapy, intravitreous steroid injection, and plasmapheresis. Considering the physiopathology of CAR syndrome, local therapy may be an option as an adjunct therapy, but it may not substitute systemic immunosuppression. The patient deferred any systemic treatment and consented only with the local treatment. Following an extensive literature search, our group was unable to find any publication about the use of Ozurdex in the treatment of CAR.

Follow-up showed anatomical and functional improvements with partial reorganization of the retinal layers on OCT, which concurs with a similar published case^(5)^. Functional improvement was documented in terms of BCVA and microperimetric changes. The patient also noticed improvement in her daily life activities and reported subjective improvement of her visual acuity and reduction of her photophobia. These symptoms indicate that functional improvement may not reflect solely a learning effect. Finally, these improvements were maintained during a 12-month follow-up, longer than the usual duration of the therapeutic effect of Ozurdex for other retinal conditions such as macular edema. As the patient started systemic treatment for the new breast cancer, we could not draw any precise conclusion about the long-lasting effects on functional improvements solely related to dexamethasone intravitreal implant.

Our patient showed visual improvement in OU even though only the OS was treated and, according to the manufacturer information, the medication induces no systemic absorption. We propose two explanations for our finding as follows: (1) fluctuations of the serum antibody levels with a consequent fluctuation of the retinal injury and/or (2) reestablishment of the immune privilege of the ocular environment after a determined time interval in relation to the cataract surgery. Despite these mechanisms, steroids are known anti-inflammatory and immunosuppressive agents, and the use of a dexamethasone implant might have also contributed to the inhibition of the retinal degeneration related to CAR.

In conclusion, the use of Ozurdex appears to be a viable alternative for the treatment of CAR by promoting probable stabilization of clinical manifestations. However, we recognize the need for further studies with larger numbers of patients and longer follow-up periods to clarify the potential usefulness of the 0.7-mg dexamethasone implant as an alternative and/or adjuvant therapy for CAR syndrome.

## References

[1] Bussat A, Langner-Lemercier S, Salmon A, Mouriaux F (2018). Paraneoplastic Syndromes in Ophthalmology. J Fr Ophtalmol.

[2] Kordyś M, Przeździecka-Dołyk J, Turno-Krȩcicka A, Misiuk-Hojło M (2018). Immunopathogenesis of ophthalmological paraneoplastic syndromes:
recent findings. Adv Clin Exp Med.

[3] Ten Berge JC, van Rosmalen J, Vermeer J, Hellström C, Lindskog C, Nilsson P (2016). Serum autoantibody profiling of patients with paraneoplastic and
non-paraneoplastic autoimmune retinopathy. PLoS One.

[4] Weleber RG, Watzke RC, Shults WT, Trzupek KM, Heckenlively JR, Egan RA (2005). Clinical and electrophysiologic characterization of
paraneoplastic and autoimmune retinopathies associated with antienolase
antibodies. Am J Ophthalmol.

[5] Irizarry FJ, Kopplin LJ, Salek SS, Adamus G, Saleh M, Biggee K (2017). Recovery of outer retinal laminations on optical coherence
tomography after treatment of cancer associated retinopathy. Am J Ophthalmol Case Rep.

[6] Suimon Y, Saito W, Hirooka K, Kanda A, Kitai H, Sakakibara-Konishi J (2017). Improvements of visual function and outer retinal morphology
following spontaneous regression of cancer in anti-recoverin
cancer-associated retinopathy. Am J Ophthalmol Case Rep.

[7] Ferreyra HA, Jayasundera T, Khan NW, He S, Lu Y, Heckenlively JR (2009). Management of autoimmune retinopathies with
immunosuppression. Arch Ophthalmol.

